# Amino Acid-Coated Zeolitic Imidazolate Framework for Delivery of Genetic Material in Prostate Cancer Cell

**DOI:** 10.3390/molecules28124875

**Published:** 2023-06-20

**Authors:** Shakil Ahmed Polash, Koen Garlick-Trease, Suneela Pyreddy, Selvakannan Periasamy, Gary Bryant, Ravi Shukla

**Affiliations:** 1 School of Science, RMIT University, Melbourne, VIC 3000, Australia; s3705029@student.rmit.edu.au (S.A.P.); s3722091@student.rmit.edu.au (K.G.-T.); s3740494@student.rmit.edu.au (S.P.); selvakannan.periasamy@rmit.edu.au (S.P.); gary.bryant@rmit.edu.au (G.B.); 2 Ian Potter NanoBiosensing Facility, NanoBiotechnology Research Laboratory (NBRL), RMIT University, Melbourne, VIC 3000, Australia; 3 Centre for Advanced Materials and Industrial Chemistry, RMIT University, Melbourne, VIC 3000, Australia

**Keywords:** metal–organic framework, amino acid, surface functionalization, biomolecule, gene delivery, toxicity, cellular uptake

## Abstract

Metal–organic frameworks (MOFs) are currently under progressive development as a tool for non-viral biomolecule delivery. Biomolecules such as proteins, lipids, carbohydrates, and nucleic acids can be encapsulated in MOFs for therapeutic purposes. The favorable physicochemical properties of MOFs make them an attractive choice for delivering a wide range of biomolecules including nucleic acids. Herein, a green fluorescence protein (GFP)-expressing plasmid DNA (pDNA) is used as a representative of a biomolecule to encapsulate within a Zn-based metal–organic framework (MOF) called a zeolitic imidazolate framework (ZIF). The synthesized biocomposites are coated with positively charged amino acids (AA) to understand the effect of surface functionalization on the delivery of pDNA to prostate cancer (PC-3) cells. FTIR and zeta potential confirm the successful preparation of positively charged amino acid-functionalized derivatives of pDNA@ZIF (i.e., pDNA@ZIF_AA_). Moreover, XRD and SEM data show that the functionalized derivates retain the pristine crystallinity and morphology of pDNA@ZIF. The coated biocomposites provide enhanced uptake of genetic material by PC-3 human prostate cancer cells. The AA-modulated fine-tuning of the surface charge of biocomposites results in better interaction with the cell membrane and enhances cellular uptake. These results suggest that pDNA@ZIF_AA_ can be a promising alternative tool for non-viral gene delivery.

## 1. Introduction

Gene therapy is an emerging field of medicine that aims to treat genetic disorders by delivering therapeutic genes into cells. The goal of gene therapy is to correct the underlying genetic mutations that cause disease and provide long-term benefits to patients [1]. Gene therapy has the potential to revolutionize the treatment of a wide range of genetic disorders, including monogenic diseases (e.g., cystic fibrosis) and complex disorders such as cancer and heart disease [2]. However, the development of effective gene delivery systems remains a major challenge. The delivery system must be able to effectively transfer therapeutic genes into cells while minimizing toxicity and off-target effects. These challenges must be addressed in order to develop safe and effective gene delivery systems for clinical applications.

The success of gene therapy depends on the efficient delivery of therapeutic genes into target cells. The cellular uptake of gene delivery systems is a critical factor in determining their efficacy and safety. Enhancing the cellular uptake of gene delivery systems improves their efficiency for gene transfer, increasing the chances of a successful therapeutic outcome. In addition, increasing the cellular uptake and specificity of gene delivery systems reduces the amount of material required for treatment, reducing the risk of toxicity and other adverse effects. This is particularly important for in vivo gene therapy, where large amounts of material may be required for systemic delivery.

Metal–organic frameworks (MOFs) are a class of hybrid porous materials that consist of metal ions linked by organic ligands. In recent years, they have gained significant attention due to their unique properties, including high stability, tunable pore size, and large surface area [3,4,5,6,7]. These properties make MOFs ideal for encapsulating therapeutic drugs and genes and improving their delivery into cells [8,9,10,11]. MOFs are synthesized by the self-assembly of metal ions and organic ligands, and the resulting structures can be tailored to meet specific needs by adjusting the composition and preparation conditions [12]. MOFs have been shown to have several advantages over traditional drug/gene delivery systems, such as viral vectors and liposomes [13,14]. Unlike viral vectors, MOFs do not have the risk of integration into the host genome, reducing the risk of adverse effects [6,15]. Unlike liposomes, MOFs have a large surface area, making it easier to conjugate with therapeutic molecules and improving their delivery [16]. Recently, an amino acid-boosted MOF biomimetic strategy has been reported for biocatalysis applications [17,18,19]. Biomineralization is a novel approach to encapsulating biomolecules including nucleic acids in MOFs [20]. It can be performed under physiological conditions and provides exceptional protection of nucleic acids by encapsulating them. A zeolitic imidazolate framework (ZIF) is the most studied MOF subclass having zeolite-like structures. Recent studies have investigated ZIFs as potential carriers for gene delivery [21,22,23,24]. It has been shown that ZIFs can effectively encapsulate genetic material, such as plasmid DNA, and protect it from degradation [10,25]. They can be functionalized with targeting moieties, such as peptides or antibodies, to enhance specificity and reduce off-target effects [26,27,28]. Additionally, the large internal surface area of ZIFs allows for the encapsulation of large amounts of genetic material, which can increase the efficiency of gene delivery. In recent times, a new phase of ZIF-8 called ZIF-CO_3_-1 (ZIF-C) has been developed which is different from typical cubic sodalite ZIF-8. The ZIF-C phase only formed when the biocomposite was prepared and washed with water [21,29]. A direct comparison between ZIF-8 and ZIF-C encapsulating nucleic acids is reported by Pyreddy et al. [29]. ZIF-C has a higher nucleic acid-loading efficiency and cell viability.

In recent years, research has focused on using amino acid-coated nanoparticles for gene delivery. Amino acids are the building blocks of proteins and play a crucial role in cellular uptake and gene delivery. Positively charged amino acids (e.g., arginine and lysine) are natural components of cell membranes and are recognized by cells as non-threatening. The amino acid coating is a common strategy to enhance the cellular uptake of nanoparticles used for drug or gene delivery [30,31]. The importance of amino acid coating lies in its ability to modify the surface properties of the nanoparticles, making them more biocompatible and increasing their interaction with cells [32,33]. The use of amino acids-rich peptides has shown great promise in nucleic acid delivery in mammalian cells [34,35,36]. Several amino acids have been used for coating nanoparticle-based delivery systems, including lysine (Lys), arginine (Arg), histidine (His), cysteine (Cys), and tyrosine (Tyr) [37]. A nanoparticle coated with amino acids is compatible with cell membranes, making it easier for the cells to uptake the nanoparticles [38,39]. Histidine is a weakly positive charge amino acid (PI 7.64) at physiological pH that has been used as a coating for nanoparticle-based gene delivery systems due to its ability to form coordination bonds with metal ions [40], enhancing stability and biocompatibility [41]. Histidine functionalization has also been employed to enhance the sensing properties of ZIF-8 [42,43]. Additionally, there have been studies that have investigated the use of amino acid-coated nanoparticles in combination with other delivery strategies, such as conjugation with targeting moieties or encapsulation of genetic material, to further enhance the efficiency of gene delivery [30,44,45,46,47,48]. The optimal choice of amino acid will depend on the specific requirements of the gene delivery system and the target cells.

The amino acid coating has several advantages for enhancing the efficiency and safety of gene delivery by MOFs. Some of these advantages include improved cellular uptake [30]; improved biocompatibility [49]; enhanced stability [33]; targeted delivery [50]; and improved efficacy [51]. The optimization of the coating conditions is critical to optimize the stability of the coating and the efficiency and specificity of cellular uptake. Optimization includes choosing the right amino acid (as different amino acids have different chemical properties and interactions with target cells) and optimizing the method of coating and the concentration of amino acids. Considerations include (1) high concentrations of amino acid may improve the stability and efficiency of the coating but may also increase the risk of toxicity and immunogenicity, while low concentrations may reduce the risk of toxicity and immunogenicity but may also reduce the stability and efficiency of the coating. (2) Longer durations of coating may provide more time for the amino acid to interact with target cells but may also increase the risk of toxicity and immunogenicity, while shorter durations may reduce the risk of toxicity and immunogenicity but may also reduce the stability and efficiency of the coating.

The use of amino acid-coated MOFs has the potential to enhance the cellular uptake and specificity of gene delivery systems, providing a safer and more effective approach to gene therapy. In this work, we present a one-pot synthesis for the preparation of nucleic acid-encapsulated ZIFs at room temperature with post-synthesis modification of amino acids into the framework structure. The biocomposites have been characterized by X-ray diffraction (XRD), Fourier transforms infrared spectroscopy (FTIR), scanning electron microscopy (SEM), and zeta potential. The cellular uptake and transfection efficiency of the biocomposites were assessed by fluorescence microscopy to visualize the expression of the delivered nucleic acid into PC-3 cells.

## 2. Results and Discussion

### 2.1. Synthesis and Characterization of Amino Acid-Coated pDNA@ZIF

**Figure 1:**
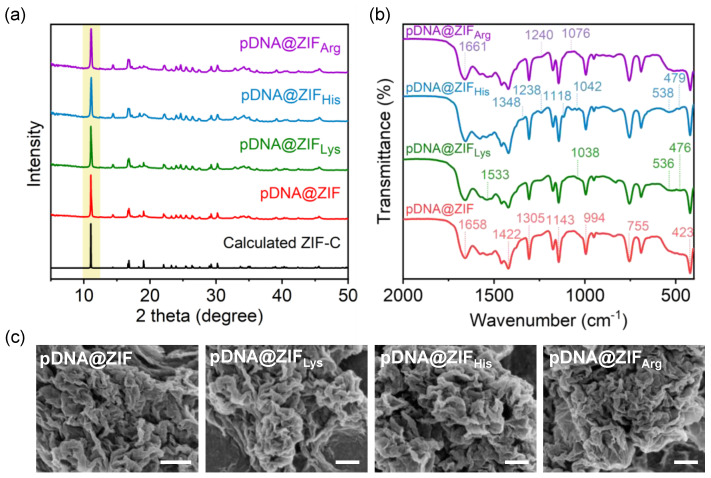
Characterization of amino acid-functionalized pDNA@ZIF. (**a**) XRD, (**b**) FTIR, and (**c**) SEM images of pristine and functionalized pDNA@ZIF. Scale bars 500 nm.

The pDNA@ZIF was synthesized by the biomineralization method at room temperature. Both the prepared pDNA@ZIF and functionalized pDNA@ZIF_AA_ were characterized to understand their physiochemical properties. The characterization of the biocomposites is shown in Figure 1. The crystallinity of the materials was studied by the XRD pattern of the dried biocomposites (Figure 1a). The diffraction profile of the as-prepared pDNA@ZIF matches with the calculated diffraction pattern of carbonate-based ZIF (ZIF-CO_3_-1 or ZIF-C) (Figure 1a, bottom trace) [52]. The presence of an intense peak (2θ = 11°) and other X-ray reflections implies a high crystallinity of pDNA@ZIF showing good agreement with our previous results [21,24,29]. The XRD patterns of pDNA@ZIF_AA_ were found to be similar to the diffraction pattern of pDNA@ZIF, revealing that the crystalline structure of the MOF composites was not affected, even after functionalizing with cationic amino acids (i.e., Lys, Arg, His). Therefore, the experimental diffractograms suggest that the amino acid functionalization of pDNA@ZIF does not impact its crystal structure. The concentration of amino acid did not influence the framework structure as at higher concentrations, the obtained biocomposite retained the same crystallinity (Figure S1). The FTIR spectrum of pDNA@ZIF was found to be similar to the FTIR spectrum of ZIF, which was previously reported [29], and confirmed the successful functionalization of amino acids on the pDNA@ZIF surface (Figure 1b). From these results, it can be concluded that amino acid is present on the MOF surface. From the FTIR spectrum of pDNA@ZIF, the peaks were at 423 cm^−1^ (Zn-N stretching), 755 cm^−1^ (aromatic C-H bending), 994 cm^−1^ (C-N bending), 1144 and 1174 cm^−1^ (aromatic C-N stretching), 1300–1450 cm^−1^ (imidazole ring stretching), and 1590 cm^−1^ (C = N stretching) [53,54]. The FTIR of the functionalized biocomposites confirms the successful coating of amino acids on the MOF surface. Two short bands at 476 and 536 cm^−1^ correspond to C-H bending vibrations of Lys in pDNA@ZIF_Lys_, 1038 cm^−1^ for C-O stretching, and 1533 cm^−1^ for symmetric bending of the NH_3_^+^ group [55]. In pDNA@ZIF_His_, the peaks for sp^2^ C-H bending vibrations (at 479 and 538 cm^−1^), C-O stretching (at 1042 and 1238 cm^−1^), imidazole group C-N stretching (at 1118 cm^−1^), and COO^-^ symmetric stretching (at 1348 cm^−1^) confirm the presence of the His molecule on the pDNA@ZIF surface [56,57,58]. In pDNA@ZIF_Arg_, the peak at 1076 cm^−1^ corresponds to the C–N stretching vibration of arginine and a broad peak at 1240 cm^−1^ refers to the C-O stretching of the Arg molecule on the ZIF surface [59].

**Figure 2:**
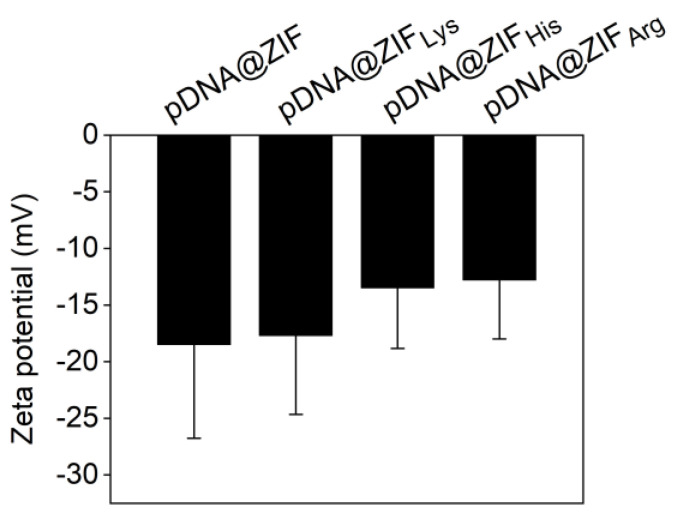
Zeta potential of the functionalized biocomposites.

The morphology of the biocomposites was observed by SEM (Figure 1c). A plate-like irregular morphology of the biocomposites was observed under an electron microscope [21,29]. The functionalized pDNA@ZIF did not show a notable difference in morphology. The zeta potential results are shown in Figure 2. As expected, the presence of the positively charged amino acids lowers the (negative) zeta potential by between 7–10 mV depending on the amino acid. The SEM of pure ZIF-C is shown in Figure S2.

### 2.2. Loading Efficiency and Protection

**Figure 3:**
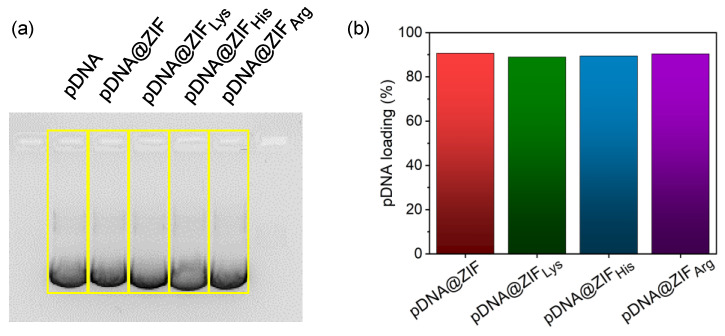
The pDNA-loading content in amino acid-modified ZIF. (**a**) Gel image of pDNA and all ZIF derivatives obtained from agarose gel electrophoresis and (**b**) calculated loading efficiency of all biocomposites using ImageJ software.

Nucleic acid-loading efficiency was determined by agarose gel electrophoresis. The obtained intensity of the DNA band signal in the agarose gel image was analyzed with ImageJ software [60]. An equal amount of pure plasmid was used as a control to quantify encapsulated nucleic acid after post-synthesis modification with amino acids. No apparent change in nucleic content was observed by modifying the ZIF shell with amino acids (Figure 3). This suggests amino acid coating does not interfere with the DNA-loading content in ZIF.

**Figure 4:**
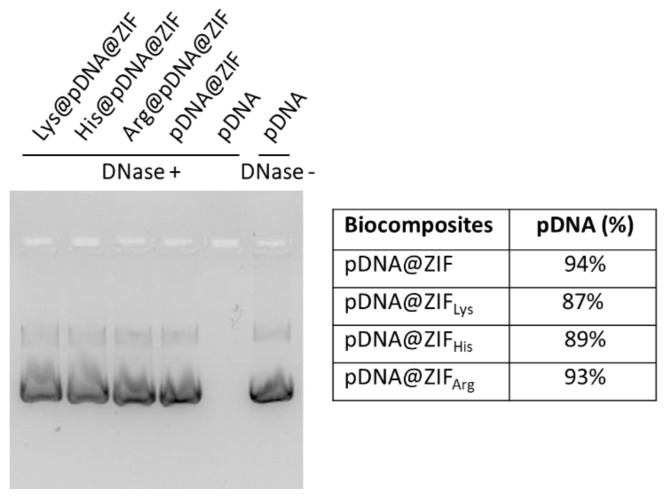
Agarose gel image showing protection of nucleic acid from DNase treatment.

For the successful and reliable delivery of nucleic acid into cells, the ideal carrier should protect the encapsulated nucleic acid from degradation by serum nucleases [22]. The stability of encapsulated pDNA against nuclease degradation was investigated with endonuclease enzyme (DNase I) to check the effectiveness of the functionalization strategy. As shown in Figure 4, DNase effectively cleaved the free pDNA in an aqueous solution. However, the pDNA@ZIF_AA_ showed good protection against the endonuclease as the encapsulated pDNA remained intact as it is in pristine pDNA@ZIF.

### 2.3. Release Profile

**Figure 5:**
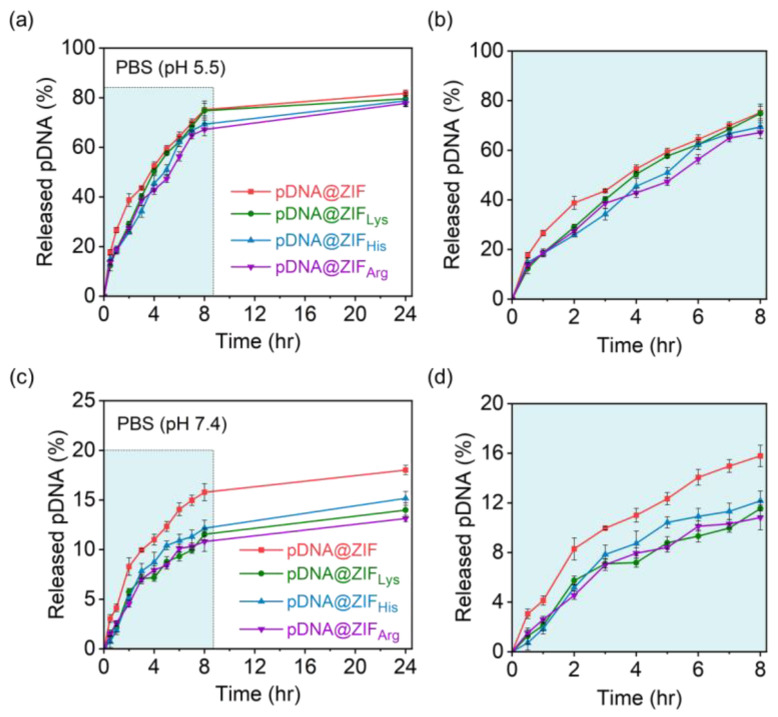
Cumulative release of pDNA from pristine and amino acid-modified ZIFs in PBS (pH 5.5 and 7.4). Total release of pDNA up to (**a**,**c**) 24 h and (**b**,**d**) 8 h in (**a**,**b**) acidic and (**c**,**d**) neutral buffers. The shaded sections (**b**,**d**) correspond to the immediate impact of the 2 buffered media on releasing the loaded plasmid in short time incubation (up to 8 h).

In the context of nucleic acid encapsulation and delivery systems, the release profile of nucleic acid from MOFs refers to the pattern or rate at which the protein is released from the MOF structure over time. When nucleic acids are encapsulated within MOFs, the MOF shell can serve as a protective environment, shielding the nucleic acids from degradation and providing controlled release capabilities. The release profile describes how the nucleic acid is released from the MOF matrix. The time-dependent release kinetics of both pristine and functionalized pDNA@ZIFs in PBS at two different pH values are shown in Figure 5. This result indicates that the pH plays an important role in ZIF stability, stimulating pDNA release under acidic conditions more than under physiological conditions. At every time point, the release rate of pDNA at neutral pH was lower than at acidic pH. A burst release of pDNA was recorded in acidic pH within 8 h (Figure 5b) and complete saturation (~77%) achieved within 24 h (Figure 5a). Since Zn-N coordination bonds are destabilized under acidic conditions, high proton concentrations in an acidic buffer (pH 5.5) trigger the release of pDNA [61,62]. However, at neutral pH, the pDNA@ZIF_AA_ showed a low pDNA release rate (<20% in 24 h) as compared to pDNA@ZIF (with no amino acids) (Figure 5c,d). This might be due to the effect of functionalization where amino acid coating stabilizes the material [63]. The release of pDNA at neutral pH is due to the affinity of the phosphate ions in the buffer with the metal, and the formation of amorphous zinc phosphate nanospheres [64]. From the drug delivery point of view, the stability of material at neutral pH is preferable as it corresponds to the physiological condition of the body wherein the material should be intact and not release the cargo. Our study also suggests that the functionalization process does not significantly affect the release of encapsulated plasmid from ZIF as it provides stabilization of the framework. In fact, release at pH 5.5 (as shown in Figure 5a,b) should prove beneficial to ensure the intracellular plasmid DNA release. Our findings confirm previous studies that polymer coatings improve the stability of ZIF-8 under acidic conditions [27]. These results indicate that the functionalization process does not significantly affect the release of encapsulated cargo from ZIF. Therefore, the data suggest that amino acid functionalization seems to be helpful for the optimal nucleic acid release profile.

### 2.4. Cytotoxicity

**Figure 6:**
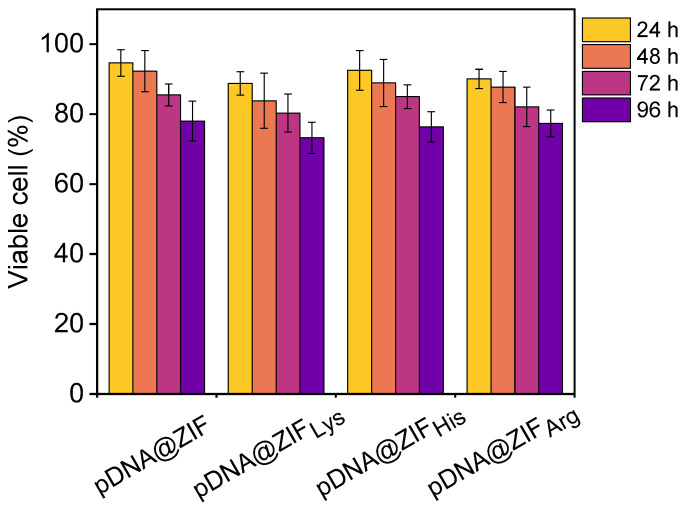
Time-dependent MTT cytotoxicity assay of pDNA@ZIF and pDNA@ZIF_AA_. Data are shown for three independent replica experiments (*n* = 3). The unpaired *t*-test was performed with a 95% confidence level and no significant change in cell viability was recorded.

To assess the effect of pDNA@ZIF and pDNA@ZIF_AA_ on cell viability, the MTT assay was performed. One of the critical requirements for MOFs to act as successful gene delivery systems is establishing their biocompatibility. Here, pDNA@ZIF and pDNA@ZIF_AA_ were used for cellular toxicity assessment at 2 different time points (i.e., 24 and 48 h) alongside AA-functionalized biocomposites. The cytotoxicity results indicated no progressive change in viability with amino acid-functionalized derivatives (Figure 6). At both 24 and 48 h of treatment, more than 80% of cells remained viable, and no significant toxicity was observed for any of the 3 pDNA@ZIF_AA_. Moreover, no notable toxicity was noticed even the cells were treated for long time with pDNA@ZIF_AA_. Cells maintained ~77% viability up to 96 h (Figure 6). Thus, the cytotoxicity results support the acceptable cell viability of pDNA@ZIF after being coated with amino acids.

### 2.5. Expression of the Delivered Gene

**Figure 7:**
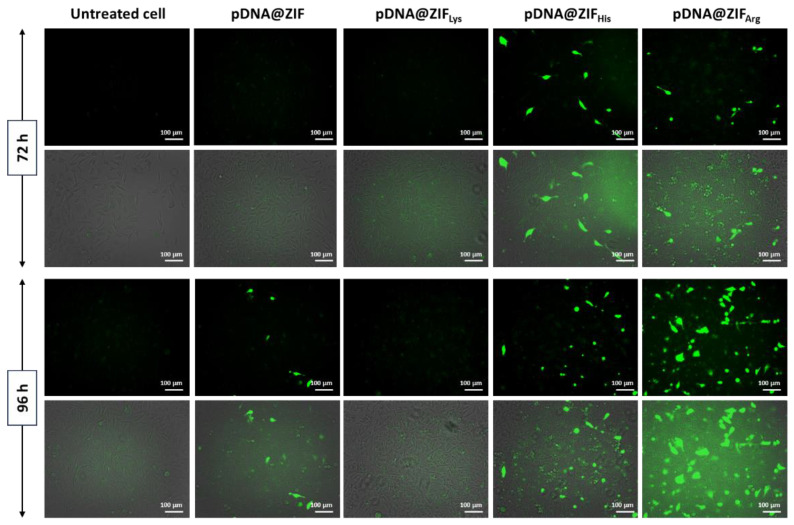
PC-3 cells were treated with pDNA@ZIF and pDNA@ZIF_AA_ for 72 and 96 h. The green fluorescence is emitted due to the expression of green fluorescent protein (GFP) from the encapsulated pDNA. Top row: fluorescence image and bottom row: merged phase contrast and fluorescence images of the treated cells.

A green fluorescent protein (GFP)-expressing plasmid (6.5 kbp) was used to assess the potential gene-loading efficiency of amino acid-functionalized ZIF. The amount of pDNA used for synthesis was chosen based on the optimal amount of DNA required for transfection [22]. The resulting biocomposites showed time-dependent expression of the delivered pDNA, with a steady increase in transfection noted after 96 hours (h) compared to 72 h of treatment (Figure 7 and Figure S3). The pDNA was only expressed at 96 h after being treated with non-functionalized ZIF. However, the His- and Arg-modified ZIF showed GFP expression at 72 h, with relatively more cells expressing it at 96 h. pDNA@ZIF_Lys_ did not show any expression. Our results so far indicate that ZIF-based biocomposites are gradually endocytosed by cancer cells through multiple mechanisms [29].

Endosomal leakage of carriers plays an important role in cargo delivery. Successful carriers must overcome endosomal vesicle entrapment and degradation to achieve the cytosolic availability of cargo biomolecules [65]. The functionalized pDNA@ZIF_AA_ possibly escapes from the endosome via the proton sponge effect and enters the host nucleus from the cytoplasm for subsequent action [66]. The protonation of the imidazole ring in 2-methylimidazole helps pDNA@ZIF to escape endosomal attack [67]. Functionalization with His enhances the therapeutic delivery of nucleic acid due to the presence of imidazole rings that influence the disruption and destabilization of cell and endosomal membranes, thereby enhancing therapeutic nucleic acid delivery [56,68]. The pDNA@ZIF_Arg_ biocomposites have the potential to enter cells very effectively due to less negative zeta potentials. A strong green signal was observed when cells were treated for 96 h. Our study supports earlier studies as L-arginine is well known to be a more efficient cell-penetrating peptide than L-lysine [69]. Histidine and arginine are well known for enhancing the cellular uptake of carrier molecules loaded with therapeutic cargo and escape from endosomal degradation. The imidazole groups of histidine and guanidine groups in arginine enhance the cellular uptake of material and undergo protonation in endosomes. This promotes the destabilization of endosomal membranes and escapes via the proton sponge effect [56,70,71]. Functionalization of carrier molecules with histidine and arginine provides a possibility of enhanced therapeutic delivery which influences the destabilization of endosomal membranes [72,73]. On the other hand, poly-L-lysine, but not lysine, has been explored in nucleic acid delivery in the past due to its favorable biodegradation property [74]. The high positive charge density of lysine causes cytotoxicity and prevents the release of plasmid DNA. Hence, lysine coating does not seem to help endosome escape activity due to the absence of secondary and tertiary amines which results in low transfection efficiency [75].

## 3. Materials and Methods

### 3.1. Required Reagents

2-methylimidazole, zinc acetate dihydrate, l-histidine, l-arginine, and l-lysine, MTT were purchased from Sigma-Aldrich (Castle Hill, NSW, Australia). Roswell Park Memorial Institute (RPMI) 1640 Medium, fetal bovine serum (FBS), penicillin-streptomycin (PS), and 3-(4,5-dimethylthiazol-2-yl)-2,5-diphenyltetrazolium bromide (MTT) were obtained from Thermo Fisher Scientific (Waltham, MA, USA). Prostate cancer 3 (PC-3) cells were purchased from American Type Cell Collection (ATCC), Manassas, VA, USA.

### 3.2. pDNA@ZIF Synthesis

Biomimetic mineralization was employed to encapsulate the 5.8 kb length pDNA according to our previously reported methods [21,29]. Briefly, 5 μg of pDNA was added in a fresh tube having 100 μL aqueous 2-methylimidazole (160 mM). Then, 100 μL aqueous zinc acetate dihydrate (40 mM) solution was added and resuspended gently. The tube was left undisturbed at room temperature. The pDNA and imidazole mixture immediately turned the solution cloudy soon after the addition of aqueous zinc acetate. Here, pDNA acts as a nucleating agent and accelerates the Zn–imidazole coordination bonds [11]. After 15 min, the particles were collected by centrifugation (13,000 rpm for 12 min), and the white precipitate was further washed thrice with 200 μL Milli-Q water each time to remove unreacted precursors. The final product was vacuum-dried to remove trace solvent and kept at room temperature until further studies.

### 3.3. Amino Acid Functionalization

An aqueous solution of positively charged amino acids (e.g., Lys, His, and Arg) was prepared in purified water. The dried pDNA@ZIF was separately immersed in 500 and 1000 μM of amino acid solutions overnight at 300 rpm. The biocomposite was separated by centrifugation at 13,000 rpm for 12 min. The pellet was washed 3 times with 200 μL of water to remove unreacted amino acids.

### 3.4. Characterization

XRD was performed in Bruker D8 General Area Detector Diffraction System (GADDS) using Cu Kα radiation (λ = 1.54056 Å) at 40 kV generator intensity and 40 mA generator current to determine the crystallinity of the prepared materials. A concentrated drop of the sample was placed on a silica wafer to make a thin film and dried completely. The obtained raw file (.raw) was converted to UXD file format using the File Exchange Program XCH (Ver. 5.0.10, 2004, Bruker AXS, Socabim, Karlsruhe, Germany) before data analysis [24]. For analysis, the collected spectra were plotted using the OriginPro 2021b software from OriginLab [76]. FTIR was carried out using a Spectrum 100 (Perkin Elmer, Waltham, MA, USA) with a Spotlight 400 attachment. Potassium bromide (KBr) was added to the dried pellet, mixed thoroughly, and transferred to the instrument sample holder. Pure KBr was run as background initially and average spectra of 128 scans were collected from the 2000–400 cm^−1^ range with 4 cm^−1^ resolution. The FEI Verios 460 L extreme high resolution (XHR) SEM was used to characterize the morphology and particle size. The diluted particle suspension was drop-casted on a surface-clean silica wafer and dried completely to remove the solvent. A 5 mm iridium coating was applied using an EM ACE600 Sputter Coater (Leica, Sydney, Australia) to enhance conductivity and vacuum durability in the SEM chamber. The accelerating voltage and current of the electron beam were 1 kV and 0.25 pA, respectively. Zeta sizer Nano (Malvern, UK) was used to determine the zeta potential of the biocomposites.

### 3.5. Loading Efficiency Assay

The pDNA loading in the amino acid-functionalized ZIF was investigated by agarose gel electrophoresis. Both pristine pDNA@ZIF and amino acid-coated pDNA@ZIF_AA_ were dissolved in aqueous EDTA (20 mM) to break the ZIF shell and release pGFP in the solution. All samples were loaded on pre-heated 1% agarose gel containing SYBR Safe stain alongside an equal quantity of pDNA used during synthesis as control. The electrophoresis was carried out in 1 X TBE buffer for 120 min at 90 V and bands were visualized in GelDoc^®^ (BioRad^®^, Hercules, CA, USA) system.

### 3.6. Protection Assay

A nucleic acid protection assay was performed using TURBO™ DNase I (Invitrogen, Waltham, MA, USA) following the manufacturer’s protocol. Briefly, 1 μL DNase (2 U/μL) was mixed with pDNA@ZIF and pDNA@ZIF_AA_ samples and incubated for 30 min at 37 °C. An inactivation reagent was added next to all the samples to inactivate the action of DNase followed by centrifugation (10,000 rcf for 10 min). The pellet was resuspended in water. Next, 20 mM EDTA was added to all samples to break the ZIF shell and release the loaded pDNA in suspension, followed by loading the samples in 1% agarose gel electrophoresis using untreated pDNA@ZIF. Electrophoresis was carried out in 1 X TBE at 90 V for 120 min and finally, the DNA bands were visualized in GelDoc^®^ (BioRad^®^, USA). The band intensity curve and area under the curve of each peak were calculated using ImageJ software. The peak percentage of each experimental band (DNase treated (+) lanes) was divided by the peak percentage of the control band (DNase untreated (−) lane) to give the relative percent of DNA quantity associated with the nano–MOF composites.

### 3.7. Release Profile

To study the impact of amino acid coating in releasing the encapsulated pDNA from the ZIF shell, the functionalized biocomposites as well as control pDNA@ZIF were incubated in PBS solutions having two different pH (7.4 and 5.5). Here, pH 7.4 and 5.5 represent the physiological condition of normal human and tumor cells, respectively. At different time points (up to 24 h), the samples were collected to check the released pDNA content from the ZIF shell by fluorescence spectroscopy (Horiba FluoroMax^®^ 4, Irvine, CA, USA). A DNA-binding dye propidium iodide (PI, 1 µg/mL) was added into each solution and incubated in the dark for 30 min. The pDNA-bound PI (pDNA-PI) gives a typical emission peak at 617 nm (ex. 535 nm). The amount of released pDNA was calculated from pDNA-PI standard curve and expressed in % compared to the initially added pDNA.

### 3.8. Cytotoxicity

Approximately 10,000 cells/well human prostate cancer cells (PC-3) were seeded in 96 well plates and incubated for 24 h in humidified 5% CO_2_ at 37 °C incubator. Later, cells were treated with pDNA@ZIF and pDNA@ZIF_AA_ in 100 µL low-serum medium (Opti-MEM, Gibco) followed by incubation at 37 °C. After 3.5 h, the treatment media was replaced with 100 µL of RPMI medium supplemented with 10% FBS and 1% PS. The cells were then incubated for 24 h and 48 h of treatment. After incubation, the media was aspirated and 100 µL of serum-free media containing MTT (0.5 mg/mL) was added. It was further incubated at 37 °C for 4 h in the dark. Next, the MTT media was aspirated carefully without disturbing purple formazan crystals. The purple formazan crystals were dissolved in 100 µL DMSO, and the absorbance was measured by a microplate reader at 570 nm with a reference wavelength of 630 nm. The percentage of cell viability was calculated by the formula: cell viability % = (absorbance in sample/absorbance in control) × 100.

### 3.9. Bioactivity

To prove the functionality of encapsulated pDNA and the suitability of ZIF in delivering genetic material in mammalian cells, a transfection assay was carried out using both pDNA@ZIF and pDNA@ZIF_AA_ [24]. Approximately 150,000 cell/well PC-3 cells were seeded in a 6-well plate and incubated for 24 h in humidified 5% CO_2_ at 37 °C incubator. Later, the medium was removed and 2 mL of fresh medium containing pGFP alone, pDNA@ZIF, and pDNA@ZIF_AA_ was incubated for 3.5 h at 37 °C. The media was replaced with a fresh complete RPMI medium and incubated at 37 °C. Following 72 and 96 h of treatment, cells were imaged using ZOE^TM^ fluorescent cell imager (Bio-Rad, South Granville, Australia) to check the expression of GFP in the treated PC-3 cells.

## 4. Conclusions

Coating cationic amino acid on a nucleic acid-encapsulated MOF shell has shown promise for carrying genetic material efficiently into cells. Functionalization improves the biocompatibility of the particles, increasing their specificity and safety for use in biomedical applications. This approach has been shown to effectively increase the efficiency of gene transfer into cells, making it a promising area of research for future biomedical applications. However, it is important to note that while amino acid coating has shown promising results in pre-clinical studies, more research is needed to fully assess its safety and efficacy in clinical applications. Thus, further studies are needed to fully understand the potential and limitations of amino acid-coated MOFs for drug or gene delivery.

## Data Availability

Not applicable.

## Supplementary Materials

The following supporting information can be downloaded at: https://www.mdpi.com/article/10.3390/molecules28124875/s1, Figure S1: XRD of pDNA@ZIF functionalized with 1000 µM of amino acids; Figure S2: SEM images of ZIF-C; Figure S3: Phase contrast images of cells.

## Sample Availability

Samples of the compounds are available from the authors.
