# Supplementary material for: Amino Acid-Coated Zeolitic Imidazolate Framework for Delivery of Genetic Material in Prostate Cancer Cell
Source: Molecules. 2023 Jun 20;28(12):4875. doi: 10.3390/molecules28124875 (PMC10302622; doi:10.3390/molecules28124875)
Supplement: Supplementary file 1 [file molecules-28-04875-s001.zip › molecules-2434258-supplementary.pdf]

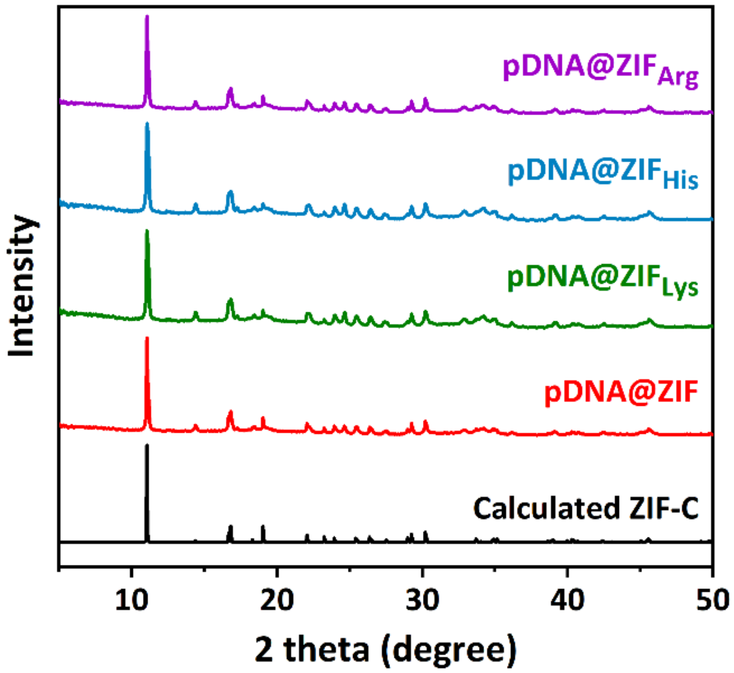

Figure S1. XRD of pDNA@ZIF functionalized with 1000 µM of amino acids.

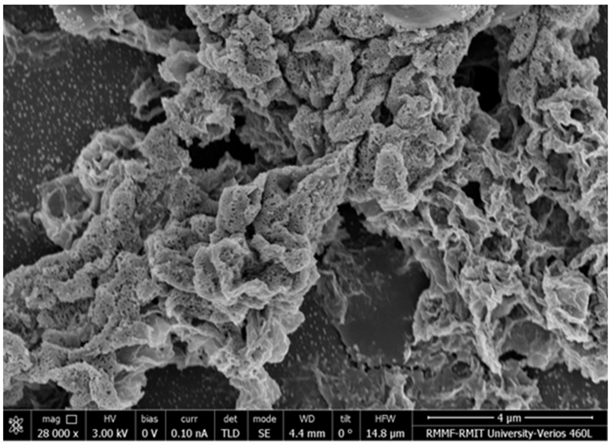

Figure S2. SEM images of ZIF-C.

Cytotoxicity: Approximately 7500 and 5000 cells/well PC-3 were seeded in 96 well plates for 72 h and 96 h treatment respectively. The cells were incubated for 24 h in humidified 5% CO_2 at 37°C incubator. The same experimental steps described in section 3.8 was followed to conduct cytotoxicity at 72 h and 96 h.

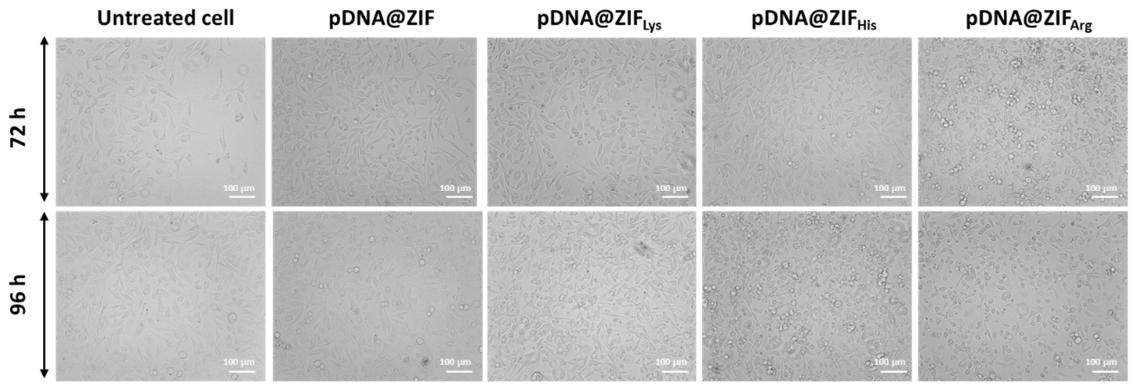

Figure S3. Phase contrast images of cells taken in phase contrast and fluorescence microscope.
